# Island Invasion by a Threatened Tree Species: Evidence for Natural Enemy Release of Mahogany (*Swietenia macrophylla*) on Dominica, Lesser Antilles

**DOI:** 10.1371/journal.pone.0018790

**Published:** 2011-04-13

**Authors:** Julian M. Norghauer, Adam R. Martin, Erin E. Mycroft, Arlington James, Sean C. Thomas

**Affiliations:** 1 Institute of Plant Sciences, University of Bern, Bern, Switzerland; 2 Faculty of Forestry, University of Toronto, Toronto, Ontario, Canada; 3 Forestry, Wildlife and Parks Division, Roseau, Commonwealth of Dominica, West Indies; Centre National de la Recherche Scientifique, France

## Abstract

Despite its appeal to explain plant invasions, the enemy release hypothesis (ERH) remains largely unexplored for tropical forest trees. Even scarcer are ERH studies conducted on the same host species at both the community and biogeographical scale, irrespective of the system or plant life form. In Cabrits National Park, Dominica, we observed patterns consistent with enemy release of two introduced, congeneric mahogany species, *Swietenia macrophylla* and *S. mahagoni*, planted almost 50 years ago. *Swietenia* populations at Cabrits have reproduced, with *S. macrophylla* juveniles established in and out of plantation areas at densities much higher than observed in its native range. *Swietenia macrophylla* juveniles also experienced significantly lower leaf-level herbivory (∼3.0%) than nine co-occurring species native to Dominica (8.4–21.8%), and far lower than conspecific herbivory observed in its native range (11%–43%, on average). These complimentary findings at multiple scales support ERH, and confirm that *Swietenia* has naturalized at Cabrits. However, *Swietenia* abundance was positively correlated with native plant diversity at the seedling stage, and only marginally negatively correlated with native plant abundance for stems ≥1-cm dbh. Taken together, these descriptive patterns point to relaxed enemy pressure from specialized enemies, specifically the defoliator *Steniscadia poliophaea* and the shoot-borer *Hypsipyla grandella*, as a leading explanation for the enhanced recruitment of *Swietenia* trees documented at Cabrits.

## Introduction

One popular and widely held mechanism for successful biological invasions is the natural enemy-release hypothesis (hereafter “ERH”; [1]). Once introduced to a new range plant species are freed from top-down population regulation by specialist enemies, namely herbivores or pathogens, thereby enhancing their competitive ability relative to native plants, which helps increase their distribution and abundance [2]. Various aspects of the ERH have been addressed in many field studies, mostly from temperate zones, but these have yielded a muddled picture, in part because few studies have examined both community and biogeographical scales of analysis [3]–[5]. Woody plant invaders of forests are being increasingly reported [6]–[13]; however, apart from the woody shrub *Clidemia hirta* introduced to Hawaii [14], there remains scant investigation of ERH as a driver of woody plant invasions in the tropics, especially for long-lived trees (but see [15]). This is surprising given that both the intensity of herbivory and degree of enemy host-specialization on trees is thought to be greater in tropical forests than their temperate counterparts [16]–[18].

One potential tropical system wherein processes associated with ERH may prevail is in introduced populations of high-value tropical timber species in the pan-tropical Meliaceae family. Because of their valuable timber, plantations of American mahoganies (*Swietenia macrophylla* King, *S. humilis* Zucc., and *S. mahagoni* (L.) Jacq.) have been repeatedly established throughout the tropics. Natural enemies are a well-known problem in mahogany: under favorable growth conditions *Swietenia* populations suffer severe, widespread attack from specialised *Hypsipyla* shoot-borers (Lepidoptera: Pyralidae), namely *H. grandella* Zeller in the Neotropics, and from host-switching *H. robusta* Moore in the Paleotropics [19]. Many Meliaceae timber species are wind-dispersed, tolerant of partial shade, survive well in disturbed habitats, and show high growth responses to increased light [18], [19], [20]–[24]: a suite of life-history traits that make them potential invaders of forests ([1], [13], [25], [26], but see [12]).

Given its extensive planting globally, it is not surprising to learn that *Swietenia macrophylla* shows natural regeneration in some places, namely islands such as Hawaii and Puerto Rico [23], [27], the Seychelles [8], as well as Sri Lanka and Trinidad [28] — but its spread beyond planted sites has never been rigorously demonstrated and it is unclear whether *Swietenia* spp. can invade adjacent habitats and possibly displace local plant species [9], [13], [28], [29]. Prior assessments of the invasiveness of mahogany have been anecdotal, and have not explicitly considered the ERH. In his compendium of plant invaders Weber [30] calls *S. macrophylla* a “fast growing and shade tolerant tree”, and lists it as an invader in tropical Asia, present but not invasive in the Mascarenes and Galapagos, and not present in the Caribbean. By contrast, Whitmore [29] considered *S. macrophylla* incapable of invading forest despite repeated and extensive planting globally.

In the present study we present empirical evidence to address whether or not *Swietenia* species are invading (or are likely to invade) Cabrits National Park (hereafter Cabrits) on the Caribbean island of Dominica, West Indies (see Appendix S1), as facilitated by processes consistent with the ERH. Initial observations at the site suggested that *S. macrophylla* and *S. mahagoni* trees planted in the 1960s have established a viable naturalized population, with progeny evading attacks by *H. grandella* and recruiting outside of planting zones. This case is compelling from a conservation and resource management perspective, in that the potential invader species (*Swietenia* spp.) remains highly threatened in forests of their native range in spite of their international trade regulated under CITES Appendix II [24], [31]. Here, we address three main questions: (1) Do *Swietenia* spp. seedlings and saplings (hereafter, ‘juveniles’) at Cabrits suffer negligible herbivory compared to native Dominican woody plant species? (2) Are planted *Swietenia* populations at Cabrits reaching reproductive maturity and successfully regenerating? and (3) Is *Swietenia* regeneration displacing native vegetation? In light of *Swietenia*'s potentially fast growth in the absence of its specialised herbivores, coupled with it being a key iconic species in sustainable forest management and conservation, we briefly discuss the potential for *Swietenia* to pose an invasion risk more broadly; identify knowledge gaps and future research and monitoring needs; and address possible conservation and management implications.

## Methods

### The Commonwealth of Dominica

In the Lesser Antilles, halfway between Guadeloupe and Martinique, lies the island of Dominica (c. 754 km^2^, 15°25′N, 61°20′W; Appendix S1) renowned for its rugged topography and lush forests. Dominica's vegetation has long attracted biological interest because it remains closer to its original state than on any other Caribbean Island [32]. Although less than 48 km long and 24 km wide, it supports four principal vegetation types: (1) forest on the lower leeward slopes characterized by a low canopy (12–15 m) and deciduous species [33]; (2) a taller transitional belt of forest beginning at c. 300 m asl with more of an evergreen component, in which herbs, vines and epiphytes appear; (3) an extensive zone of mesophytic vegetation, between c. 450 and 800–1000 m asl, consisting of >20 m tall evergreen trees in a very wet environment (tropical rain forest); (4) short stature (3–6 m tall canopy) dwarf or elfin woodland restricted to the highest elevations and dominated by *Clusia mangle* (>800–1000 m asl; [33]). Precipitation is strongly seasonal; it is wettest from July to October, with dangerous tropical storms most frequent in August and September. Dominica's position in the Caribbean makes it highly vulnerable to Atlantic hurricanes — notably David in 1979, and most recently Dean in 2007 — which are undoubtedly the most important natural disturbance to its fauna and flora. Just north of Dominica, long-term data (1635–2000) was put together for Guadeloupe, where strong hurricanes of at least a category II, III, or IV occurred on average every 4, 8 and 13 yrs (respectively) [34].

Harrison [35] provides an excellent account of Dominica's early land use history and economic development. In the mid 20^th^ century, ‘Big-leaf mahogany’ (*Swietenia macrophylla*) and ‘Small-leaf’ mahogany trees (also known as ‘West Indies’ mahogany, *Swietenia mahagoni*) were planted on the island in at least five places. One of these planting sites was Cabrits, a peninsula that is now protected as a national park in the northwest of the island. Henceforth, mahogany (or just *Swietenia*) will be used to denote both these two tree species unless otherwise indicated. Unfortunately the historical records of mahogany's planting in Cabrits were lost when Hurricane David, a Category 5 storm, passed over Dominica in 1979; however, trees were planted in lines, facilitating relocation of surviving trees from the initial plantings.

### Cabrits National Park in Dominica

Established in 1986, Cabrits National Park, at 531 ha, is the smallest of three national parks on Dominica, and protects rare flora and fauna of regionally threatened moist semi-evergreen tropical forest. Because Cabrits is located at low elevation on the leeward side of the island it receives <2000 mm of rain per annum, typically 1300–1700 mm per annum. The Park encompasses a peninsula and surrounding marine area near the town of Portsmouth (Appendix S1). Prior to 1986 humans repeatedly cleared the vegetation at the Cabrits peninsula, most notably when it was home to Fort Shirley, which was abandoned in the mid 19^th^ century, but whose ruins can still be seen today. Hence the present vegetation at Cabrits is largely the product of succession over the last 150 years. A few small-scale farmers occupied the land just prior to when plantations were established. Some of the existing network of historic paths and trails were re-opened over the years beginning around 1982.

### Mahogany's native range, conservation status and biology

Mahoganies are canopy-emergent forest trees reaching heights of 30–50 m and stem diameters of 1–3 m [20]. Like other high-value Meliaceae (e.g., closely related *Khaya* and *Entandrophragma* spp. from central African forests; [22]) they form elegant, straight boles whose wood is prized for its strength, rot resistance, workability, and appearance [24]. Although light demanding when small, they can tolerate floods, hurricanes, and fire disturbances when mature [20].

*Swietenia mahagoni* is native to the southern Florida peninsula and the Greater Antilles, whereas *S. macrophylla* has a far greater range, from Mexico through Central America into South America (Columbia, Peru Ecuador, and Brazil; [20], [24]). Both of these *Swietenia* species are on the IUCN Red List. Where accessible, mahogany populations have been effectively ‘mined’ in their native forests, most of which have become fragmented and converted to agriculture and ranching after logging [20], [21], [31]. In spite of CITES protections — *S. mahagoni* was listed in 1992, and *S. macrophylla* added in 2003 — *S. macrophylla* is still widely harvested in an unsustainable manner [31], [36], while *S. mahagoni* has long been “commercially extinct” [19], [20], [24].

*Swietenia macrophylla* is a fast-growing emergent tree (≥1 cm yr^−1^ diameter increment under favorable conditions), but its seedlings and saplings require at least small-scale canopy openings to reach maturity [20], [21], [31], [37], [38]. Mahoganies do not form seed banks and are not ‘pioneers’ (*sensu* [39]); as adults they are long-lived and seeds germinate and seedlings establish best under shaded conditions [40]. Adult trees are deciduous, shedding their leaves during the dry season, at which time woody fruit capsules dehisce their valves and prevailing winds disperse the winged propagules [20], [41]. Onset of *S. macrophylla* fruiting as a function of tree size is highly variable in both Brazilian and Mexican forests, but generally becomes more frequent in trees >70 cm diameter [37], [38], [40].

In mahogany's native range, the well-known *H. grandella* shoot-boring caterpillars reduce juvenile tree growth rates during leaf flush events by hollowing out the growing stem and damaging new leaves, thereby negatively affecting wood quality in both mixed and monoculture plantations; their impact in natural forests is less clear [18] (but see [42], [43]). *Hypsipyla grandella* attacks many Meliaceae host species [19], [20]; another more highly specialised enemy caterpillar, *Steniscadia poliophaea* Hampson (Noctuidae), defoliates new leaves of seedlings and saplings of *S. macrophylla* in natural and logged forests in its Brazilian range [40], [41], [44]–[46].

### Survey of mature mahogany stands at Cabrits

In February 2008 we located and mapped planted mahogany stands in Cabrits, as well as park trails and other points of interest using a global positioning system (Garmin GPS 76*CS*x); we searched for possible adults, defined as trees ≥20 cm dbh or with distinctive bark morphology associated with maturity (Appendix S1). In the eastern portion of the park, we discerned six planting zones consisting of three blocks with regular spacing of mature *Swietenia* trees, and three zones where it was not (hereafter, ‘J’, ‘T’ and ‘Z’). Among the ‘J’ zone was the only buttressed tree found (45.2 cm diameter at 1.3 m height, hereafter ‘dbh’). We measured dbh for a subset of mahogany trees and labeled them with aluminum tags for future measurements. For 2 of the 3 planting blocks (dark red, Appendix S1), only those trees forming the perimeters were measured (hereafter ‘A’ and ‘B’ blocks), equivalent to sampling a c. 5-m wide swath, whereas in patches ‘T’, ‘Z’, and ‘J’ all trees (*n* = 29, 14, and 12, respectively) were tagged and measured. To assess reproduction in the population, we used binoculars to search for presence/absence of fruit capsules in the crowns of all measured trees in the ‘A’ and ‘B’ blocks plus those in patches ‘Z’ and ‘J’, but not ‘T’ (total sample of trees surveyed, *n* = 103).

### Herbivory and *Swietenia* size-class distribution surveys

To evaluate regeneration and leaf damage on mahogany juveniles relative to common native woody species, in March 2008 a network of 37, 5-m-fixed radius circular plots (each 78.5 m^2^; total area = c. 2900 m^2^) was established in the eastern portion of Cabrits (plots denoted by circled ‘×’ gray symbol in Appendix S1). Using three separate start locations on trails in each of the three main planting blocks, a plot center was established ≥20 m perpendicular to trails. Moving roughly parallel to trails along the North-South axis, plots were added at intervals of 25–40 m, such that distances between plots' centers were ≥15 m to ensure sufficient park coverage and spatial interspersion among plots. Seven sampling plots were in the three mahogany blocks, leaving 30 outside of these three high-density planting areas. Plot centers were marked with PVC pipes driven into the ground, and radii with flagging tape on four cardinal directions. Because of the network of crossing trails, where required, plots were adjusted to be ≥15 m from trails. To estimate the local canopy cover and light environment, a hemispherical photo was taken at each plot center at a height of 50 cm using a Nikon Coolpix 4500 affixed with an FC-E8 fisheye converter lens (Nikon, Tokyo, Japan). Photos were analyzed by one person using Gap Light Analyzer v.2 software [47] to obtain percent forest canopy openness and estimated daily photosynthetic photon flux density (PPFD; mol m^−2^ d^−1^).

In each plot all live mahogany stems ≥30-cm in height were counted, tagged, and measured (hereafter, ‘juveniles’). Of these juveniles, 1–12 mahogany saplings (defined here as ≥1 cm dbh) closest to the plot center were surveyed for herbivory; if saplings were lacking then smaller-sized ‘seedlings’ were measured (mean and median mahogany *n* per plot = 5 and 4.75, respectively). Comparable measurements were made on max 10 juveniles representing nine common native and one other introduced woody plant species (10 non-mahogany species, Table 1). In each plot we aimed for at least one representative individual of each of the 10 species, but more often than not only 1–3 juveniles of fewer species were available. On tall plants the most accessible leaves were sampled, but on shorter plants with multiple branches accessible, the highest two branches were sampled. Leaf damage was scored visually by same person (JMN) on all leaves present or up to maximum of 10 leaves per individual, per species (5 leaves per branch, or max 10 per single stem). To further minimize bias, the most recent mature leaves were scored for the amount of leaf area damaged by insects and/or fungal pathogens. Leaf damage was quantified as one of the following seven herbivory classes: (1) ≤1%, (2) 5%, (3) 10%, (4) 25%, (5) 50%, (6) 75%, and (7) 90%. All mahogany and non-mahogany juveniles were measured for dbh ≥1 cm with calipers; or for smaller juveniles, height and root collar diameter.

**Table 1:** List of common woody plant species surveyed for standing levels of leaf damage in March 2008 at Cabrits National Park, Dominica in the Lesser Antilles.

Species	Family	Origin	Habit
*Tabebuia heterophylla*	Bignoniaceae	native	tree to 20 m height
*Chrysobalanus icaco*	Chrysobalanaceae	native	shrub/tree
*Morisonia americana*	Conneraceae	native	tree to 10 m height
*Lonchocarpus benthamianus*	Fabaceae	native	shrub/tree to 4 m height
*Ocotea coriacea*	Lauraceae	native	shrub/tree to 6 m height
*Eugenia ligustrina*	Myrtaceae	native	shrub/tree to 7 m height
*Eugenia* sp.	Myrtaceae	native	NA
*Myrcia citrifolia*	Myrtaceae	native	shrub/tree
*Pimenta racemosa*	Myrtaceae	introduced	tree to 13 m height
*Pisonia fragrans*	Nyctaginaceae	native	tree to 14 m height
*Swietenia macrophylla*	Meliaceae	introduced	tree to 30–50 m height
*Swietenia mahagoni*	Meliaceae	introduced	tree to 30–50 m height

Habit corresponds to descriptions provided by [62] for the non-*Swietenia* species. For *Swietenia* habit see Methods section for references on its stature and biology. NA: not available.

### Tree community surveys to assess competitive displacement

To assess the evidence for native species displacement by mahogany, in May 2010 we made additional surveys of tree seedling composition in 26, 5-m radius plots (total area 2041 m^2^). These were set up on the same eastern portion of Cabrits where herbivory and size-class distribution sampling was carried out. However, due to difficulties in relocating the 36 original survey plots of 2008, these stem diversity plots did not overlap completely with the original network of plots. Plot centers were located ≥20 m apart along linear transects, and placed to ensure spatial interspersion of sampling points. Within each plot all woody stems <50 cm in height were identified to species and counted. For analyses stems were categorized as either “*Swietenia*” or “native”. In 21 of these plots (total area, c. 1648 m^2^) diameters of all live stems ≥1 cm dbh were measured, and individuals identified to species.

### Statistical analysis

Species differences in standing herbivory levels were analyzed using an unbalanced mixed-model ANOVA on log-transformed percent damage, with plot included as a random factor. To avoid pseudo-replication, we averaged leaf damage per individual for *S. macrophylla* and the 10 native species (Table 1), and again at the plot level prior to analysis. Additionally, because no significant differences in leaf damage were found between *S. macrophylla* saplings (≥1-cm dbh) and seedlings (*t*-test, assuming unequal variances, d.f. = 51, *P* = 0.687), these data were pooled for analysis. Post-hoc comparisons of Least Squares means were based on 10 *t*-tests adjusted for multiple comparisons using a sequential Bonferonni correction, because we were not interested in testing all possible pair wise comparisons, just the specific contrasts planned between *S. macrophylla* vs. 10 other species under ERH (Table 1). Comparisons of *S. macrophylla* herbivory rates between the study site and areas within its native range were based on a one-tailed *t*-test assuming unequal variances on log-transformed percent leaf damage. One-way ANOVA was used to compare stem size distributions among planted sites, and here an unplanned post-hoc comparison of group means was made using Tukeys HSD. Tree size at onset of maturity was estimated for *Swietenia* as the inflection point of a modified logistic regression function describing reproductive status as a function of dbh [48]. Due to a marginally significant decline in probability of reproduction among the largest trees in the sample, a second-order term was included in the model, and the inflection point calculated using a numerical approximation with 95% confidence limits generated by bootstrapping (500 simulations). For community displacement analyses, stems were categorized as “*Swietenia*” or “native”, and stem basal area (BA) was calculated as BA = π*r* ^2^, where *r* is stem radius (dbh/2).

## Results

### Herbivory and insect damage

**Figure 1:**
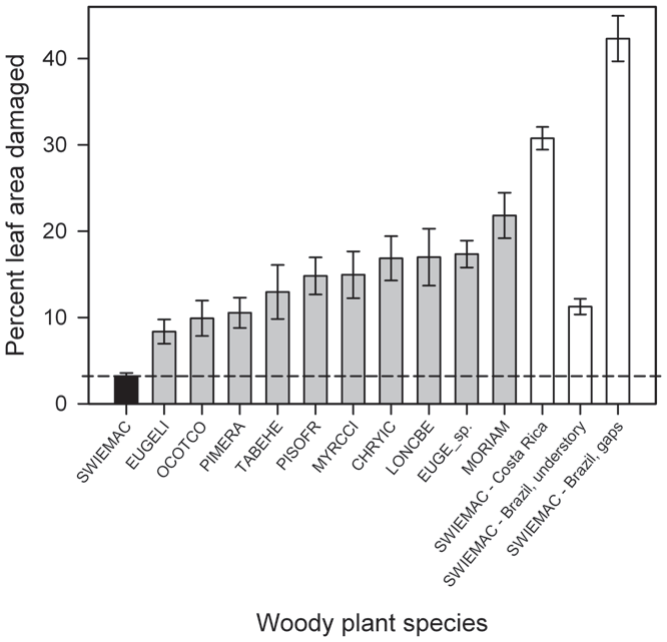
Mean leaf herbivory on juveniles of the introduced big-leaf mahogany (*Swietenia macrophylla* = SWIEMAC, the black bar) and 10 co-occurring woody plant species in Cabrits National Park, Dominica. Not shown because of too small a sample size is introduced small-leaf mahogany (*S. mahagoni*), which had an average (± SE) of 9.65%±3.14 (*n* = 4 plots). Sample sizes for the 11 species bars were from left to right: 20, 14, 30, 22, 9, 25, 11, 19, 9, 25, and 17 plots, respectively. For comparison, in white bars at the far right, are shown mean (± SE) levels of herbivory for planted seedlings in semi-evergreen and deciduous secondary forest in Costa Rica (calculated from *n* = 18 data points in Figure 2c in [61]), for *n* = 304 wild seedlings in the understory of an unlogged Amazonian forest [44], and for 168 seedlings planted in *n* = 14 small canopy gaps in the same forest [45]. Species are listed in Table 1.

A total of 262 non-*Swietenia* plants in the 37 plots were found and surveyed for leaf damage, for a total of 2573 leaves. A total of 753 simple and/or compound leaves from 95 *Swietenia* juveniles were also surveyed. Nearly all juveniles were *S. macrophylla* (89), with only six *S. mahagoni* surveyed (from four plots). Mean standing leaf damage was significantly lower on *S. macrophylla* than all 10 other co-occurring species surveyed at Cabrits (unbalanced mixed-model ANOVA on log-transformed percent damage, with plot as the random factor, *P*<0.0001, *F*_10, 180_ = 9.22; planned post-hoc comparisons of *Swietenia* vs. 10 native species using sequential-Bonferroni corrected α; Fig. 1). *Swietenia macrophylla* had a median leaf damage of 2.6% (min, max: 1.0% and 6.7%, *n* = 20 plots), and *S. mahagoni* a median leaf damage of 4.2% (min, max: 3.2% and 13.9%, *n* = 4 plots), while all other species experienced a median leaf damage ranging from 6.4% to 20.9%, *n* = 9–30 plots per species; Fig. 1). This level of herbivory on *S. macrophylla* was significantly lower than that recorded on leaves in natural and secondary forests in its native range (three unbalanced *t*-tests: Cabrits vs. Costa-Rica, *n* = 38 plots, d.f. = 23.67, *t* = 18.91, *P*<0.0001; Cabrits vs. Brazil-sun leaves, *n* = 34 plots, d.f. = 27.53, *t* = 20.19, *P*<0.0001; and Cabrits vs. Brazil-shade leaves, *n* = 393 individuals, d.f. = 260.97, *t* = 4.15, *P*<0.0001; Fig. 1).

Leaf damage on *Swietenia* was not significantly influenced by plot distance to the nearest *Swietenia* adult (Pearson *r* = −0.10, *P* = 0.65), a result that also supports ERH. In *S. macrophylla*'s native Brazilian range, leaf herbivory by *Steniscadia poliophaea* caterpillars is strongly distant-dependent, declining rapidly with increased distance to the nearest *S. macrophylla* tree [45], [46]. In contrast, at Cabrits, *Swietenia* leaf damage was only significantly influenced by light availability, having a non-linear asymptotic or possibly hump-shaped relationship (%damage = −4.57+3.03 PPFD−0.2628 (PPFD^2^) with an apparent peak in damage at c. 6 mol m^−1^ d^−1^ (polynomial regression; adj. *r* ^2^ = 0.31, *F*_2, 18_ = 5.01, *P* = 0.02). The light environment of sampling locations (*n* = 36, one plot not recorded) had a mean (± SD) percent canopy openness of 8.2±2.4, and a PPFD of 5.4±1.81 mol m^−2^ d^−1^. Taken together, our evidence suggests that what little herbivory occurs on *S. macrophylla* at Cabrits is mainly driven by variation in light availability, and not distance to nearest adult-sized conspecific.

Only 20 of 190 *Swietenia* juveniles ≥1-cm ≤5-cm dbh (10.5%) had ‘top-dead’ hollowed out stems, and six more stems had other symptoms of stem boring (13.6% attacked at most). Very little forking or lateral branching was observed in saplings, which generally maintained their apical dominance. *Hypsipyla grandella* shoot-boring reduces *S. macrophylla* growth rates and densities in plantations in its native range [18]–[20], [42], [43], [49]. Due to infrequent shoot-boring we could not test if it was density- and/or distance-dependent.

### *Swietenia* size-class distributions and regeneration in Cabrits

**Figure 2:**
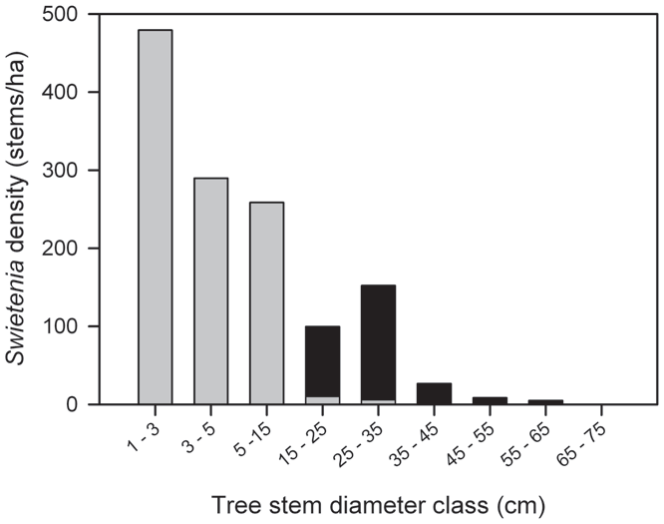
Histogram showing the size-class distribution of introduced mahogany (*Swietenia*) in Cabrits National Park, Dominica. Trees ≥20 cm dbh were sampled in planting zones (*n* = 137 trees: 127 *S. macrophylla*+10 *S. mahagoni*) and stems ≥1 cm dbh (*n* = 285) were sampled in a network of small 37 circular plots (see Appendix S1). In the latter, 15 stems >20 cm dbh were also found most of which were from plots in the planting zones (Appendix S1). To standardize sampling effort, derived counts for each sampling scheme are expressed here on a per hectare basis. The black bars indicate size-classes or portions thereof that most likely originated through planting efforts in 1960's to early 1970's, whereas gray bars indicate natural regeneration inferred from the pattern of planting. The latter includes four trees in the 15–25 cm dbh class found in plots outside planting zones, as well as four ‘X’ trees (22.6, 28.4, 29.9 and 30.5 cm dbh) found in our general survey of planting zones (see Appendix S1).

Analysis of the reproductive survey yielded an estimated size threshold for reproduction of 25.1 cm dbh (CI_95_ = 21.2, 29.0 cm; *n* = 103 trees of which 22 were reproductive: 17/94 *S. macrophylla* and 5/9 *S. mahagoni*) at Cabrits, and this estimate is applied in interpreting further results. In surveys of planted stands 133 mahogany trees ≥20 cm dbh were found at Cabrits. Of these, 127 were identified as *S. macrophylla* and 10 as *S. mahagoni*. Overall, most *Swietenia* individuals in the planting zones at Cabrits were in the 25–35 cm dbh size class (Fig. 2). But the size-class frequency distributions were not similar across the 5 planted areas (one-way ANOVA, *F*_4, 136_ = 8.44, *P*<0.0001). Among the planting blocks surveyed, block ‘J’ had significantly larger trees on average (± SE) at 40.6±3.6 cm (*n* = 12), than the other four planted areas (Tukeys HSD post-hoc test, *P*<0.05), and probably were among the very first trees planted at Cabrits. Moreover, the largest ‘J’ tree found (58.5 cm dbh) was twice the diameter of the smallest ‘J’ tree (25.4 cm). The mean sizes of *Swietenia* for the planting blocks ‘A’ and ‘B’ and patches ‘T’ and ‘Z’ (means: 27.1–29.2 cm dbh; *n* = 28, 49, 30 and 18, respectively, with four additional trees 18.8–19.8 cm dbh included) were not statistically different (Tukey HSD post-hoc test, *P* values>0.5). However, in all areas but the ‘Z’ patch a few large adults could be found (>40 cm dbh, *n* = 7).

A group of likely 1^st^ generation (‘X’) trees (*n* = 4) was found south of the ‘Z’ patch (see Appendix S1; 22.6, 28.4, 29.9 and 30.5 cm dbh). Although these stems exceeded the critical reproductive size threshold, two of the four clearly lacked the distinctive brown sheath barking of mature trees and instead had the grayish, smooth bark with vertical striations characteristic of fast-growing non-reproductive individuals (*personal observations*).

In addition to a reproductively viable plantation cohort, we also found evidence for successful establishment of a second-generation understory and mid story *Swietenia* cohort. Of the 286 *Swietenia* stems ≥1 cm dbh found in 37 plots, 271 (c. 95%) were non-reproductive juveniles <20 cm dbh in size, of which nearly all were *S. macrophylla* (255, or c. 94%) whose abundance peaked in the 1–5 dbh size class (Fig. 2). Stem densities of juvenile *Swietenia* at Cabrits were extremely high, ranging from 15 to 64 per 5-m-radius plot, or c. 0.2 to 0.8 individuals m^−2^. This result is consistent with ERH, as one would never find such a high density of *Swietenia* saplings within the seed shadow of parents in forests in its native range (e.g., 0.0174 seedlings m^−2^, 0.0004 m^−2^ stems 2.5–10 cm dbh, in Bolivia [37]; 0.0–0.0093 m^−2^ stems 1–10 cm dbh in Pará, Brazil [50]).

**Figure 3:**
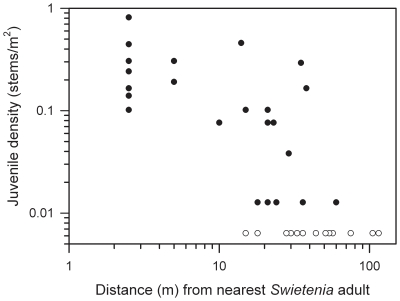
The relationship between density of introduced mahogany (*Swietenia*) juveniles in sampled plots (*n* = 37) and distance to the nearest potential parent source (≥20 cm dbh) in Cabrits National Park, Dominica. Note that the original counts per 78.5 m^2^ plot are expressed on a per m^2^ basis, and both axes are shown on a log-log scale. To show those plots with zero counts of mahogany stems (white symbols) we assigned them the same arbitrary value. The number of juveniles, including stems <1-cm dbh but >30-cm tall, totaled 305, and were made up almost entirely of *Swietenia macrophylla* (c. 97%) with very few *S. mahagoni* (10 juveniles). There was a significant, sigmoidal relationship between log (density +1) and the log (distance) transformed variables (non-linear regression, *F*_2, 34_ = 22.6, *P*<0.0001, adj. *r* ^2^ = 0.55, equation: y = 1.41/{1+exp[(−x+1.18)/0.263]}.

As expected, juvenile *Swietenia* densities declined with distance from the nearest potential source tree (defined as ≥20 cm dbh) following a sigmoidal function (Fig. 3), but were not significantly associated with either percent canopy openness or PPFD (linear regression, *P* = 0.48 and quadratic regression *P* = 0.19, respectively, *n* = 37 plots). This highlights the importance of seed limitation in restricting the extent of mahogany's recruitment outside of planting areas [7], [13], [15].

### Native displacement by *Swietenia*

**Figure 4:**
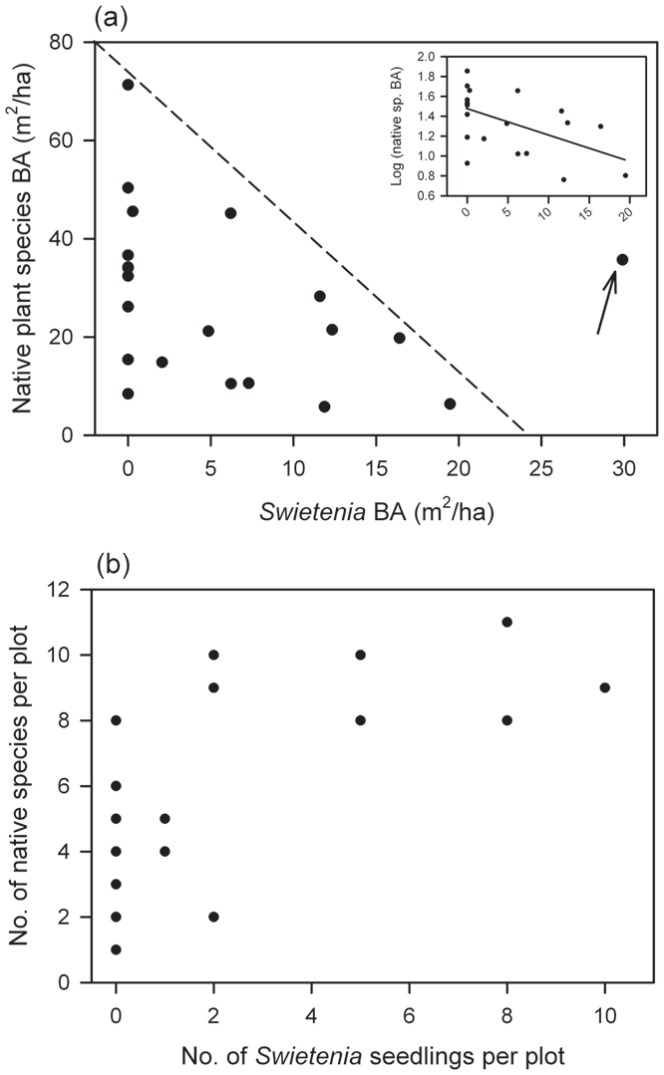
Relationships between native woody plant abundance and diversity to the abundance of introduced mahogany (*Swietenia*) in Cabrits National Park, Dominica. Basal area is expressed on a per ha basis (**a**), and native plant diversity as a function of mahogany abundance is for stems <50 cm tall (**b**) in respectively *n* = 26 and 21 plots (respectively) at Cabrits National Park, Dominica. In (**a**) the arrow points to an unusual statistical outlier in the data set, and the dashed lined indicates a hypothesized upper boundary to native species recruitment as mahogany's local dominance increases. All plots were each 78.5 m^2^ and surveyed in May 2010. The inset in (**a**) shows a negative relationship between the log-transformed basal area of native species and that of mahogany (with the outlier omitted). In (**b**), although seven data points are visible, in fact there are 16 out of 26 plots with zero (‘0’) mahogany seedlings present because of duplicity in y-values.

A weak negative correlation was found between *Swietenia* and native species BA (r = −0.392; Spearman rank test, *P* = 0.0787, *n* = 21; Fig. 4a). With a statistical outlier removed, (see arrow, Cook's D = 3.0–3.5) a linear regression was marginally significant (*F*_1,18_ = 5.33, *P* = 0.033, *r* ^2^ = 0.23), but stronger when native species BA was log transformed to properly satisfy regression assumptions (*F*_1,18_ = 6.65, *P* = 0.019, *r* ^2^ = 0.27, see Fig. 4a inset).

In contrast to the results for basal area of larger stems, there was a positive relationship between seedling abundance of *Swietenia* and that of native woody plants (Fig. 4b). Again, linear regression was not possible, but a quadratic fit indicated that in areas with more *Swietenia*, more native species also occurred (Fig. 4b). When grouped into plots with vs. those without *Swietenia* ≥1-cm dbh present (*n* = 10 vs. 16 plots, respectively), on average twice as many native species (7.4±0.93 SE) grew where *Swietenia* also did than where it was absent (3.0±0.52; *t*-test assuming unequal variances, *t* = 4.2, d.f. = 14, *P* = 0.001).

## Discussion

### Enemy release hypothesis

Our results agree with key predictions of the enemy release hypothesis (ERH), and are also supported by a lack of distance-dependent herbivory, suggesting that generalist herbivores are responsible for the negligible leaf damage to *S. macrophylla* observed at Cabrits. *Swietenia* shows high rates of natural regeneration at the site, with second-generation individuals approaching reproductive maturity. However, the evidence for negative effects on native tree diversity is ambiguous, with a weak negative correlation between *Swietenia* basal area and local species basal area of native taxa at larger size classes, but a positive relationship between *Swietenia* seedling density and local species richness of native seedlings.

Documentation of relaxed enemy pressure and an attendant increase in *Swietenia* performance, relative to abundant native co-occurring woody species, is both unusual and novel. The only comparable field study of ERH for tropical forest trees found that percent leaf damage was not associated with invasive spread of introduced species from a botanical garden in Tanzanian humid forest [15]. In his review of case studies, Nair [18] concluded that *S. macrophylla* suffers *equal if not greater damage* in introduced monoculture plantations outside its native range than within it (p. 142). In native forests, however, *S. macrophylla* is less vulnerable to *H. grandella* attacks than in plantation settings, probably due to higher densities of predatory and parasitoid wasps, lower conspecific stem densities, greater shading, and more foliage interference with host searches [18], [21], [42], [43], [50]. The low incidences of shoot-boring, and a complete lack of *H. grandella* visual confirmations at Cabrits, suggest the absence or near-absence of this specialized predator. In line plantings of *S. macrophylla* in the Peruvian Amazon, *H. grandella* attacks ranged from 12% to 81% of stems [43] and in southeastern Amazon forest, they ranged from 1.9% to 35.9%, but increased with size of the canopy gap opening made [21]. In Mexican plantations with comparable stem densities to Cabrits, the mean proportion of attacked individuals ranged from 10% to 74% (median = 49%) [49].

In a broader context, aspects of the ERH have been tested more frequently in temperate zones on trees and smaller-sized plants. For example, generalist enemies had a greater impact on introduced spruce (*Picea* spp.) than natives in the introduced range (France), contrary to ERH (ref. [28] in [2] review paper). More recently, in support of ERH, leaf herbivory on Norway maple (*Acer platanoides*) was greater in North America than in its native European range (1.6% vs. 7.4%, respectively) [51]. A review of studies that compared herbivore load and/or damage in both native and introduced ranges found support for ERH in 5 of 7 cases [4], but among these, only DeWalt et al. [14] quantified percent leaf area missing. By contrast, community level comparisons of congeners in the introduced range have yielded only equivocal support for ERH [4], [5]. In particular, one large common garden experiment in Ontario using 15 pairs of old-field plant species found that introduced species had slightly more leaf damage than natives, not less as predicted by ERH (7.5% vs. 5.3%, on average) [52]. An earlier review similarly found conflicting results between biogeographical and community studies of enemy release [3]; perhaps, in part, because no study reviewed was conducted on the same host plant at both scales (as done here). In fact there are almost no manipulative studies that examine population dynamics in the context of ERH. Ours is among a very few (in any system) to not only have examined impacts both in the native and introduced range, but also at the community level.

Although using both a community and biogeographical approach strengthened inference from our observational evidence, it provided only conditional support for ERH. For example, our static sampling of leaf damage, while a good proxy for chronic herbivory, precludes any potentially important insect herbivore outbreaks. Unlike using rates of herbivory, it also missed any leaves consumed entirely by herbivores or abscised prematurely from severe damage; however, surveyed *Swietenia* leaf nodes lacking leaves were extremely rare. Finally, in our surveys, we overlooked any native plants possibly excluded by herbivory, and thus risked underestimating levels of damage to natives. A stronger way to show that this mechanism of natural enemy release was operating at Cabrits would have been via experimentation in the field; for example, by adding seedlings of both *Swietenia* and natives, and manipulating their exposure to herbivores by physical or chemical means to assess their impact on vital rates (plant growth and survival) [2]–[5]. An excellent example of such an approach is the study of the introduced Chinese tallow tree (*Sapium sebiferum*) in Texas which found support for ERH [53]. Further, replicating such an experiment in *Swietenia*'s native range would strengthen any inferences [2]–[5], as done by DeWalt et al. [14] for the invasive neotropical shrub *Clidemia hirta* (only) which had five times more leaf damage in its native than introduced range (Costa Rica and Hawaii, respectively). An extreme manipulation would introduce the natural enemy from the native range into multiple populations within the introduced range, and observe plant population responses (i.e., biocontrol, but see [2]). While in general desirable, experiments are not always feasible. In our case, several reasons for why are (1) Our research was within a protected area where insecticides are forbidden, as was mesh netting on aesthetic grounds. (2) There are strict (appropriately so) controls on species introductions in Dominica. (3) Observing demographic responses to introductions would require many years for trees.

### Invasive risk posed by mahogany

Reduced herbivory and elevated *Swietenia* seedling densities did not diminish native species diversity at the seedling stage (Fig. 4b), although at later life stages (≥1-cm dbh) *Swietenia* likely continued to escape herbivory and might have begun to displace native vegetation (Fig. 4a). In the absence of experimentation, establishing whether or not native declines in abundance or diversity are caused by *Swietenia* proliferations will require vegetation surveys that move beyond static to dynamic patterns. One reason for why we cannot definitively state that *Swietenia* is invading vis-à-vis native displacement may be a matter of the spatial and temporal scales at which our study was conducted. Any displacement trend of *Swietenia* on native vegetation may only be observable at larger spatial scales of analysis, and/or over longer time-periods [9], [13], [23]. The ∼50 years since planting at this site has allowed only one cohort of trees to reproduce. To speculate, over longer time frames, ERH processes observed at Cabrits may allow *Swietenia* to spread further if current growing conditions experienced by seedlings are similar to conditions in which trees reached reproductive status. Canopy openness at Cabrits (8.2%) was comparable to closed semi-evergreen forests of S. *macrophylla*'s native range (mean ± SD, 6.3%±1.7 and 7.1%±0.51 from [44], [45] respectively); however, a lower canopy height at Cabrits may facilitate more light transmission to *Swietenia* seedlings and saplings [21] enabling them to persist longer under shaded conditions (e.g., [23]).

Current knowledge of mahogany suggests that in the absence of substantial shoot-boring and herbivory on its leaves, *S. macrophylla* juveniles should perform optimally in terms of elevated growth and reduced mortality rates [18], [21], [37], [40], [44], [45], and may thus attain maturity sooner [54]. Indeed, a crucial first step for any organism to become a potential invader in a novel environment is to survive long enough to reach reproductive maturity. *Swietenia macrophylla* trees in their native range tend to reach reproductive onset at ∼20–30 cm dbh [37], [38], [40]. Augmenting published accounts, we estimated reproductive onset of *Swietenia* at Cabrits to be 25.1 cm dbh (*sensu* [48]). At Cabrits many *Swietenia* trees, including some first-generation recruits, exceed this threshold, and are acting as a seed-source for local establishment and/or future invasion. However, repeated surveys of reproduction, seedling establishment, and tree growth are needed to properly document demographic trends. Investigating other planting areas in Dominica outside of Cabrits is therefore recommended; for this could also serve to test whether natural *Swietenia* regeneration has occurred beyond 100 m of planted parent trees (Richardson [13]) and, where permissible, to confirm the ERH mechanism using enemy-exclusion experiments. For example, on Dominica *S. macrophylla* has also been planted in rain forests within the Central Forest Reserve and at Laudat, which more closely resembles Amazonian seasonal rain forest in its southern native range.

### Conservation and management implications

It is vital from a park management perspective to know whether or not an introduced species will have an ecological and/or economic negative impact [1], [11], [12], [27], [55] lest limited resources be wasted needlessly for costly eradication that serves little purpose [56], [57]. The removal of large, potentially highly fecund trees could offset these costs easily if the wood can be sold, and funds earmarked for park management and conservation purposes. Nevertheless, future *Swietenia* recruitment might not imperil protected native flora at Cabrits if additional mechanisms of coexistence are in place [9]. For example, vertical partitioning of the light gradient through the forest canopy (e.g., [58]) may well allow *Swietenia* to establish yet not fully displace native flora. Conceivably, many shrubs and small trees could survive beneath *Swietenia* crowns, or under a *Swietenia*-dominated canopy.

In this respect, the work by A. Lugo on introduced trees in Puerto Rico is particularly instructive. On that island, new forests dominated by naturalized species — namely the African tulip (*Spathodea campanulata*), White siris (*Albizia procera*), and Rose apple (*Syzygium jambos*) — have formed on abandoned agricultural lands and have facilitated the establishment of many native and endemic understory species [9]. Like stands of African tulip, young and old plantations of *S. macrophylla* and *Pinus caribeae* also helped restore native biodiversity by providing key ecological functions, in the form of a ‘nursery’ canopy and litter fall, for re-colonization of less stress-tolerant native plants at human-disturbed sites — understory species richness of natives in a 50 yr old *S. macrophylla* plantation was close to that found in paired secondary forest of a similar age [59]. Hence, the view that introduced trees are detrimental to local flora (and fauna) need not always apply everywhere: indeed, the context of each case is crucial to keep in mind.

### Conclusion

On the island of Dominica, at Cabrits *S. macrophylla* has naturalized and escaped its specialist moth herbivores, and, consistent with the ERH, the results together suggest that this is a major factor in its rapid spread at the site. In terms of its status as “invasive”, our study is consistent with results from prior work on planted *Swietenia* elsewhere in the tropics. For example, Richardson [13] remarked that after 30 years on the Galapagos Islands, introduced *S. macrophylla* had not yet shown “signs of invasive spread”, but cautioned that this status might change with time. In our view, the population of *S. macrophylla* at Cabrits is currently at an intermediate stage of the invasion process [*sensu* Richardson et al. [60]). Canopy trees have long generation times, and thus may invade habitats slowly relative to other organisms [13] — nevertheless, projecting how fast the *S. macrophylla* population is growing is possible, and recommended, but will require building a formal demographic model (e.g., [61]). Therefore, monitoring *Swietenia* populations at Cabrits and similar sites is crucial to collect needed dynamics data and to identify future negative impacts on the island's native flora and fauna [56]. This may be particularly prudent in Dominica, where canopy openings from hurricanes might further promote *S. macrophylla* regeneration in the absence of top-down control from its specialist caterpillar enemies.

## Supporting Information

Appendix S1 A map showing the location of the study site (Cabrits National Park, Dominica) with a description of the introduced mahogany planting zones and the network of sampling plots.
(PDF)
