# Supplementary material for: Island Invasion by a Threatened Tree Species: Evidence for Natural Enemy Release of Mahogany (Swietenia macrophylla) on Dominica, Lesser Antilles
Source: PLoS One. 2011 Apr 13;6(4):e18790. doi: 10.1371/journal.pone.0018790 (PMC3076449; doi:10.1371/journal.pone.0018790)

APPENDIX

A map of Cabrits National Park in Dominica showing the main ascending West trail (yellow) that begins near the site of British Fort Shirley and the relatively flat East trail (blue) and its ascending extension (purple). Two patches of mahogany trees, mostly of *Swietenia mahagoni* (i.e. green double-tree symbols), were planted along the main West trail (yellow). However, because they showed signs of severe crown damage, likely from exposure to fierce winds, these patches went unstudied. On the main East trail (blue) planting was more extensive but limited to low lying flat areas, and no adult mahogany trees (green single tree symbols) were seen along or crossing the ascending East trail (purple). Within three large blocks of mahogany adults, composed primarily of *S. macrophylla*, tree spacing appeared more-or-less regular and uniform (dark red perimeters). A pair of irregular mahogany patches in a very open, previously burnt area of the park was found midway along the main East trail. Locations of individual trees in these two patches ('T' and 'Z' trees in main text) are shown by green singletree symbols on map. We cannot, however, be sure that 100% all of these trees were planted (see Results). Finally, a row of 12 *Swietenia* trees, among the largest encountered, were found along the East trail (line of single green trees beside blue trail; 'J' trees in main text).

The locations of the 37 circular sampling plots (5-m radius) are shown by the gray encircled X symbols. The red triangle shows the location of a fruiting *S. mahagoni* tree, c. 14 cm dbh. The map was made using Garmin MapSource software v. 6.15.6 (Garmin Ltd.) using GPS derived coordinates for illustrative purposes.

Appendix

Location of study site (Cabrits National Park) on the island of Dominica in the Lesser Antilles in Central America

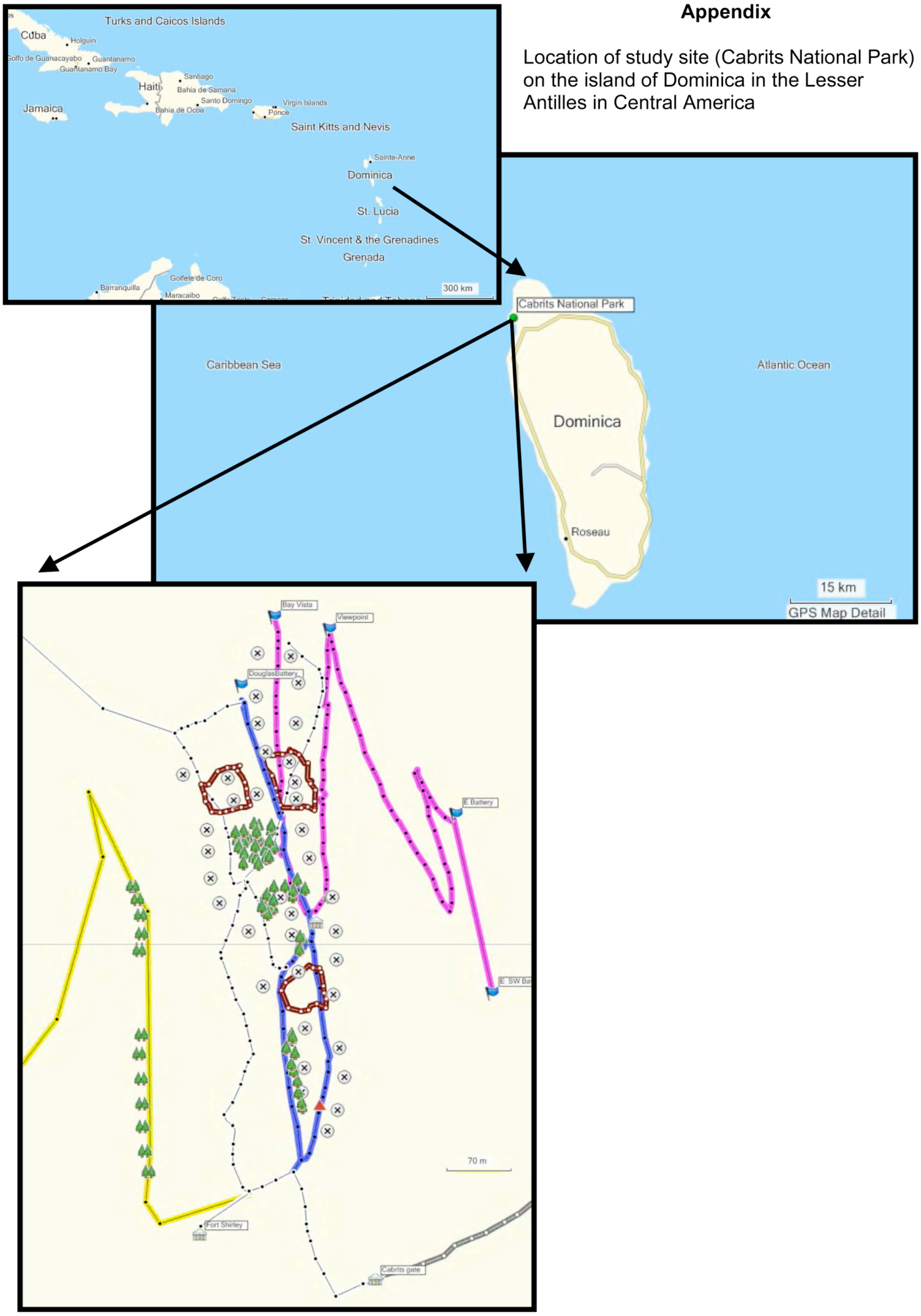

Supplement: Appendix S1 — A map showing the location of the study site (Cabrits National Park, Dominica) with a description of the introduced mahogany planting zones and the network of sampling plots. (PDF) [file pone.0018790.s001.pdf]
